# Spanish Students’ Categorical Perceptions of Feminist Movements

**DOI:** 10.1007/s10936-024-10091-8

**Published:** 2024-05-29

**Authors:** Antonio Manuel Ávila-Muñoz

**Affiliations:** General Linguistics, Facultad de Filosofía y Letras, Universidad de Málaga, Campus de Teatinos, 29071 Málaga, Spain

**Keywords:** Feminist movements, Feminism, Students, Shared perceptions, Lexical associations

## Abstract

Identifying a society’s perceptions and, by extension, opinions of a certain social movement can help to understand to what extent the movement has been successful in effecting change. When working to gain such an understanding, a focus on the student population is essential, as their opinions provide insight into the future conditions of society and, thus, into whether the movement has been successful in effecting lasting social change. The present work focusses on the feminist movements and, in line with the above, analyses the perceptions held by a sample of 600 Spanish students enrolled in compulsory secondary, pre-university, and university education. The method employed begins with the use of association tests to extract lexical networks. Then, following a theoretical transformation, the traditional lexical availability index is applied in combination with fuzzy set theory to the sample of lists obtained so as to map the structure of the collective network, a novel approach that results in different levels of compatibility. The highest levels of compatibility reveal the prototypical conceptualisation as well as the sample’s shared cognitive perception. The results suggest that although the population under study may have absorbed the feminist movements’ messages of equality and respect, distorted perceptions could still remain in certain groups analysed. This work therefore recommends that education centres may wish to consider communicating objective information on the feminist movements specifically to women, as this could ultimately lead to all students fully embracing a feminist awareness distanced from extreme ideologies.

## State of the Art

The feminist movements of today have been shaped by over a century of fighting for equal rights between men and women. Their historical development can be interpreted as a series of overlapping waves, as different currents have emerged over time that have focussed on new dimensions, such as social class, racist ideologies, and diverse gender identities (Amorós & de Miguel Álvarez, 2007; Freedman, 2003; Glucksmann, 2008; McDonald, 1997; Shields, 2016). However, in general, since the eighteenth century, when awareness first arose of the oppression of women in enlightened society, feminist movements have been defined by the social, political, and legislative nature of their demands. Today, at least in ‘Western’ societies, men and women do have equal rights, but even in these societies an egalitarian social consciousness has still not yet been established. This came to light particularly during the COVID-19 pandemic, for example, which highlighted many inequalities within the home environment that are detrimental to women (Collins et al., 2020; Dunatchik et al., 2021; Mooi-Reci & Risman, 2021; Pedersen & Burnett, 2022).

Nevertheless, overall, feminism is still a diverse phenomenon, and it has been studied from many viewpoints including that of linguistics (Gupta & Sinha, 2005; Irigaray, 1985; Kramer, 2016; Valls, 2002; Velasco Martínez, 2016; Weis et al., 2018). The results identified not only strong links to philosophy (Irigaray, 1974; Wittig, 1979) but also that the different groups within the movement as a whole can be deemed to represent approaches to politics that occasionally have associated themselves with other highly ideological political and social movements (Touraine, 2005), thereby causing other groups in society to reject their endeavours (Lazar, 2005; Nájera, 2010; Willis, 1984). In certain cases, the demands made by feminist movements even clashed with cultures and traditions deeply rooted in society (Amorós Puente & Posada Kubissa, 2007; Bajri, 2022; Botello-Peñaloza & Guerrero-Rincón, 2017; Ghuman, 2001; Medina, 2007; Nasution, 2016).

Such difficulties may well be a cause of concern for the major international organisations, who support feminist movements and also take action themselves, encouraging the right of any human being to be considered a natural person and holder of rights, irrespective of their sex or gender identity (UN Women, www.unwomen.org/en). Their expectation is surely that gender equality will help to bring an end to gender-based violence, one of the most devastating human rights violations existing in the present day. Similarly, national institutions of today’s democratic societies place great emphasis on communicating the importance of social justice and on actively creating equal opportunities and equal rights, yet according to some research their efforts are not always successful (Acker et al., 1983; Singh, 2007; Walker & Thompson, 1984). Both Duncan (2010) and Fitz et al. (2012) have drawn attention to the low degree of commitment to feminist movements among university students in today’s democratic societies, despite the fact that these students acknowledge the important role such movements play in the historical development of democracy around the world. However, ultimately, intersectionality theory indicates that not only gender affects how individuals experience society, as the various identity and societal categories like gender, race, social class, and sexual orientation overlap, combining to shape the individual’s experience (Crenshaw, 1991). It would seem that an individual cannot be reduced to one single identity and that various forms of oppression and privilege overlap.

Based on the current context, research into the perceptions held by the younger generations regarding the feminist movements is crucial. The conditions in future society depend on the attitudes of these individuals, and the fact that they are still students means research can identify any misconceptions or distorted perceptions they glean from their academic environment. In this way, research can help education promote equality where required to ensure conditions result in more equitable societies in future (Cann, 2019; Caro Blanco, 2008; de Miguel Álvarez, 2008; Esteban Galarza & Távora, 2008; Hlavka, 2014; Liberia Vayá et al., 2015).

## Aims and Research Foundations

The main objective of the present work was to discover Andalusian students’ collective perception of the phenomenon of feminist movements. Accordingly, I employed a novel approach to analyse a sample of 600 Andalusian students from three different levels of education: final year of compulsory secondary education (*Educación Secundaria Obligatoria*), final year of high school (*Bachillerato*), and final year of a bachelor’s degree (*Grado Universitario*). Building on this main aim, the premises underlying this work, described below, allow for several more specific objectives:

(A) To observe how sociocultural factors affect the perception held by the study sample regarding the phenomenon of feminist movements.
(B) To study the vocabulary associated with the phenomenon.
(C) To study the cognitive prototypes present in the collective mental representation produced by the study sample for the phenomenon.
(D) To reflect on the perceptions held by the different groups considered regarding the phenomenon.
(E) To identify any perceptions that have been recently acquired by society or may even be discriminatory towards present-day social movements seeking to achieve gender equality.

Hence, this work contributes significantly to analysing social perceptions of the feminist movement by addressing a well-researched problem using a novel approach that embraces the principles of reproducibility, replicability, and reliability. First, reproducibility is ensured by transparency and completeness when describing the methodology employed. Each step of the experimental process—from sample selection to data analysis—has been documented in detail, which will allow other researchers to faithfully reproduce the study and verify the results obtained. The methodology can be transferred to different population samples with the confidence that the results will be scientifically valid. Then, replicability is ensured by applying consolidated statistical techniques and using sufficiently large samples to guarantee the generalizability of the results. In addition, the analyses allow us to evaluate the consistency of the findings in different conditions and contexts, confirming the reliability of the obtained results, complementing those achieved to date by previous works. Finally, in the future the reliability of the study, reflected in the meticulousness of the data collection and analysis, will make guaranteeing the use of validated and reliable instruments to measure the variables of interest possible. The research procedure used serves to minimize possible biases and errors observed in prior studies.

Accessing the collective perception of a major phenomenon in today’s world presents difficulties both in theory and methodology. Nevertheless, the fields of cognitive, psycho-, and sociolinguistics at least offer a strategy for accessing such shared information: association tests, or tests of lexical availability. These can provide a detailed understanding of cognitive processes that, when combined with the novel approach employed in the present work, allow a researcher to construct collective prototypes for a society. As is well known, within the theory of categorisation (Rosch & Mervis, 1975; Taylor, 1995), the concept of *prototype* refers to an element that represents a category. They reflect the most typical or average instances of the category and serve as a mental reference point, summarising the most important features shared by the majority of category members. For example, a prototype for the category ‘fruit’ could comprise features like ‘is edible’, ‘has seeds’, and ‘is cultivated’. Some fruits like apples or oranges could be seen as more prototypical representations of the category than others. In the present work, collective prototypes are constructed by employing fuzzy set theory. The cognitive processes underlying the approach can be interpreted as collective models of perception, thereby allowing the measuring of a community’s tolerance, inclusivity, and acceptance of social developments (Ávila-Muñoz, 2022; Ávila-Muñoz et al., 2020, 2022). The following premises provide the foundations for this approach:

1. A community’s collective perception of a contentious social reality becomes apparent in lexical-semantic differences. An individual’s various social conditions (gender identity, age, public or private education, social status, profession, etc.) can affect their perception and ideology, and the effects become apparent in the lexical structure of semantic content.
2. Lexical prototypes allow researchers to determine cognitive prototypes. The individuals’ thematic vocabulary is structured around prototypical nuclei that encompass the most frequently used and shared lexical units. Frequently shared lexical units identified during analysis can be quantified and operationalised to reveal frequently shared concepts. The compatibility of vocabulary with the most central point of the lexical prototype can be parameterised by applying fuzzy set theory (Ávila-Muñoz & Sánchez Sáez, 2014), and the resulting levels within the fuzzy set reveal the cognitive prototype that reflects a society’s opinions.
3. Thus, researchers can identify stereotyped or discriminatory prototypes as well as lexical and cognitive gaps within fields of experience relevant to contemporary social life and positively impact vulnerable groups of individuals. In this context, researchers can then offer recommendations, such as for the improvement of educational curricula (regarding inclusion, tolerance, diversity, equality, transversality, etc.).

Collective perceptions and opinions are stored, articulated, and to an extent shaped by the lexicon of a language and its varieties. In representation of these collective perceptions, researchers can use lexical networks to access conceptual networks and thereby social value systems (including beliefs, attitudes, and evaluations of, as well as differences between, social groups, ethnicities, nationalities, genders, etc.) (Brugman & Lakoff, 1988; Gilquin, 2008, 2022). By focussing on the virtual lexicon, in which units are organised into thematic networks, researchers can access these social value systems for representative samples of a community without the individual speakers being able to self-monitor their responses (Schuetze & Weimer-Stuckmann, 2010). If researchers understand the degree to which a society is (un)knowledgeable about a sensitive issue, they can evaluate how much it is (dis)informed—a factor that could affect the community’s attitude towards future developments regarding the issue under study as well as others. To reach such conclusions, researchers must first access the thematic vocabulary of individuals within sample groups from the speech community or communities considered, then analyse the lexical networks available to these individuals, and finally identify the perceptions and underlying community-held beliefs (MacLaury, 1991).

The procedures used herein for the tests of lexical availability were based on those for association tests, and they are widely used in both studies on lexical availability (Ávila-Muñoz, 2016a, 2016b, 2017a, 2017b, 2017c, 2018; Ávila-Muñoz & Sánchez Sáez, 2011; Ávila-Muñoz & Villena Ponsoda, 2010) and lexical variation within a speech community (Crespo Miguel & Frías Delgado, 2008; Villena Ponsoda et al., 2018). Databases obtained using this methodology in the past have proven useful and relevant. For the present work, the data was then processed using a novel approach to produce the shared categorisation and reveal the collective prototype for the population under study, after which the results were analysed and conclusions drawn.

## Obtaining Community Prototypes. Moving from Lexical Availability to Lexical Compatibility

Studies on lexical availability were first conducted in the mid-1950s within the area of lexicostatistics, the aim being to provide an alternative to dictionaries of lexical frequency. Such dictionaries present the vocabulary used most frequently by speakers of a language in their communication. To obtain this vocabulary, basic lexical counting methods are used to determine which words occur most often, regardless of the communicative situation. However, despite the value of this type of dictionary within the area of second language teaching (Davies, 2005; Davies & Gardner, 2009; Lonsdale & Le Bras, 2009), researchers cautioned that these lists contained a high number of non-thematic words (conjunctions, prepositions, articles) and only a low number—or even no occurrences at all—of thematic words (mainly nouns), which, though frequently used, are less likely to occur in general, due to being closely linked to specific topics. For instance, many speakers will be familiar with words such as ‘plumbing’, ‘masonry’, ‘partition’, and ‘quote’, but they will only use these words when discussing the subject of house renovation.

Research on lexical availability was therefore initiated to complement dictionaries of lexical frequency within the area of second language teaching (Gougenheim et al., 1954). To access available vocabulary, researchers developed a methodology that focusses on a centre of interest—or specific topic (such as house renovation)—to access the available vocabulary expressly for that topic. The centre of interest serves as a stimulus in an association test, or test of lexical availability, during which each research participant spends a pre-defined amount of time writing down a list of words they feel are related to the stimulus (i.e., related to house renovation). The methodology then entails analysing all the word lists furnished by the sample of individuals to determine the lexical availability index (0–1) of each word provided. This index reflects frequency and position, assigning the highest values (values closer to 1)—and thus the highest relevance—to the words that appear most often (frequency) in the first positions on the lists (position).

In more recent decades, reflection on the deeper meaning of the lexical availability index has given rise to the theoretical framework of lexical compatibility. This framework assumes that when individuals produce lists of available vocabulary, they first provide words that are more prototypical, nuclear, or relevant within the centre of interest (e.g., for the centre of interest ‘fruit’: ‘apple’, ‘orange’, ‘banana’, ‘strawberry’) and then other words located increasingly further away from this prototypical nucleus (e.g., for ‘fruit’: ‘pear’, ‘melon’, ‘watermelon’, ‘tomato’, ‘pepper’, ‘cucumber’, ‘courgette’, ‘aubergine’, ‘pumpkin’, ‘avocado’). Within this framework, the lexical availability index serves as an excellent indicator of the degree of prototypicality of words in their centre of interest.

The availability of a word essentially corresponds to its accessibility within the centre of interest. The initial assumption is that each centre of interest revolves around a prototype that represents the topic (Prototype theory: Wittgenstein, 1953; Rosch, 1978; Lakoff, 1987). When an individual takes part in a test of lexical availability, they first access the nucleus of the lexical network for the prototype referenced in the centre of interest (e.g., for ‘fruit’: ‘apple’). Starting from this access point, they then make their own way around the lexical network, and this journey is reflected in the order of the words on the list they produce.

The word order on an individual’s list can therefore be interpreted as an approximate representation of their access to their personal vocabulary. Of course, obtaining the actual structure of each participant’s personal lexical network would be impossible. Such structures are not only determined by many uncontrollable biographical factors but are also dynamic and influenced by the individual’s interaction with their environment, meaning they are in a state of continuous change. Nevertheless, when applied to a sample of lists instead of an individual list, the lexical availability index produces a quantitative estimate for the structure of lexical compatibility within a centre of interest for a population. Although it is still lexical availability and the lexical availability index that are being employed, the terms lexical compatibility and lexical compatibility index are used when describing results based on a sample of lists.

Accordingly, the theoretical framework for the concept of lexical compatibility can be described as follows:

1. The centre of interest indicates an access point to a network of lexical elements perceived by the individual as interrelated and related to the concept referenced.
2. The structure of this network is subjective and inherent to the individual, meaning it cannot be directly extrapolated to other individuals.
3. For various reasons, including both social and cultural factors, individuals have similar lexical structures. When several lexical structures for a single centre of interest are combined, they produce the shared sociocultural metastructure for that centre of interest. In other words, the structure for a centre of interest does not exist in itself but is rather produced by a group of speakers. A change in the group of speakers would therefore most likely produce a change in the structure for the centre of interest.
4. When an individual is asked to provide a list of available vocabulary, they first access lexical elements closest to the most nuclear, central point of the prototype that represents the centre of interest (e.g., for ‘fruit’: ‘apple’, ‘orange’, ‘banana’). They then move through their lexical network towards words that are further from this initial access point, first providing more accessible elements (e.g., ‘pear’, ‘melon’, ‘watermelon’) and then less accessible elements (e.g., ‘tomato’, ‘pepper’, ‘cucumber’).
5. The word order on the lists plays a role in determining the structure of their compatibility within the centre of interest.
6. The structure of a centre of interest is considered to consist of a nucleus accessible to all speakers, and a periphery available to individuals depending on various factors (including their lexical capacity). The structure of compatibility within the nucleus determines the structure of compatibility throughout the rest of the spectrum.

Simply put, lexical compatibility builds on a model of linguistic representation in which lexicon is perceived to be constructed by speakers. When speakers belong to the same speech community, they share a large part of the vocabulary of their language for the reasons mentioned. It is the lexicon hub for a centre of interest that is available to all the individuals within a speech community (e.g., for ‘fruit’: ‘apple’, ‘orange’, ‘banana’, and ‘strawberry’; but not ‘pear’, ‘melon’, ‘watermelon’, ‘tomato’, ‘pepper’, cucumber’, ‘courgette’, ‘aubergine’, ‘pumpkin’, or ‘avocado’), meaning the basic vocabulary they share comprises the centre of the lexical spectrum of every centre of interest. The words that are repeated most often in the highest positions on the lists—and thus most compatible with the most nuclear point of the shared metastructure—represent the shared community prototype and determine the structure of the centre of interest. If one calculates the index of lexical compatibility (ILC, interval 0–1) for each word provided by participants, one can map the structure of the vocabulary for a centre of interest in terms of lexical compatibility. The precise mathematical foundation for the ILC is available in Ávila-Muñoz and Villena Ponsoda (2010).

Broadly speaking, the ILC can then be parameterised by incorporating fuzzy set theory (Zadeh, 1965; Zimmermann, 2001). Fuzzy sets are a generalisation of traditional set theory in which the compatibility of elements with the concept represented by the set is considered, instead of their belonging to the set. By employing a specific mathematical formula or tool offered by fuzzy set theory, different levels of compatibility or prototypicality can be established between elements and their set.

The tool that can be employed is either the FEV (Fuzzy Expected Value) or an updated but in this case more or less equal version of the FEV called the WFEV (Weighted Fuzzy Expected Value). These determine typical degrees of compatibility for fuzzy sets. Using either tool, one can establish an initial limit for the degree of belonging (e.g., for the set produced for ‘fruit’: this would most likely include at least ‘apple’) as well as further parameters for differentiating between elements that are initially still ‘very typical, or compatible’ (e.g., for ‘fruit’: this might describe ‘pear’, ‘melon’, and ‘watermelon’) and then ‘not very typical, or compatible’ (e.g., for ‘fruit’: this might describe ‘tomato’, ‘pepper’, ‘cucumber’, etc.). These two tools essentially provide several objective cut-off marks, resulting in a number of superior and inferior levels within the fuzzy set (cut-off sets or levels); each cut-off mark balances the valuation of the set of elements already selected and analysed by the tool with the degrees of compatibility of additional elements. The procedure for using lexical availability to establish degrees of belonging and construct a fuzzy set is explained in more detail in Ávila-Muñoz and Sánchez Sáez (2014).

In other words, under this approach, various lines of conceptual categorisation certainty can be established. These initially comprise the words closest to the core of the structure for the centre of interest (e.g., for ‘fruit’: ‘apple’, ‘banana’, ‘pineapple’). As the index of lexical compatibility decreases and the words are thus located increasingly further from the centre, they are also located ever closer to what one could call the line of uncertainty, beyond which a participant may question whether the word they have provided does in fact correspond to the stimulus (e.g., for ‘fruit’: ‘pepper’, ‘pumpkin’, ‘avocado’).

The incorporation of fuzzy set theory therefore allows the researcher to determine the relevant vocabulary for a centre of interest in an objective manner, and the parametrisation process ultimately allows the various compatibility levels to be described on a gradient from ‘very representative’ to ‘hardly representative’—the most compatible level being the most representative.

In sum, by translating classical lexical availability into lexical compatibility, we, as researchers, achieve the following:

1. We create a model, based on a suitable mathematical tool, that represents the situation under study and provides information that is easy to interpret.
2. We produce a flexible evaluation framework that can be applied to new data at any time.
3. We are poised to align the framework of a study with the situation it intends to analyse.
4. We obtain the cognitive prototype that represents the categorisation shared by a sample of individuals regarding a certain concept and we achieve this using an objective and explicable approach. The cognitive prototype consists of the lexical elements presenting the highest ILCs. To facilitate comparison depending on the results obtained, these may be contained within the most compatible level, constitute the entirety of the most compatible level, or constitute the most compatible level as well as additional, less compatible levels.

## Methodology

In order to obtain the available vocabulary required as the starting point for this work, a sample of students (N = 600) from the provinces of Malaga and Cadiz, Spain, were asked to take part in tests of lexical availability. The distribution of the sample is presented in Table 1. As described in the Introduction, the methodological procedures for the tests were based on those for association tests and are firmly established within the fields of socio- and psycholinguistic research, where they have proven incredibly useful, despite the simplicity with which they allow linguistic information to be obtained.

**Table 1:** The sample

	Malaga		Cadiz	
	Public centres	Private/semi-private centres	Public centres	Private/semi-private centres
*Secondary and pre-university education*				
Compulsory secondary education	50	50	50	50
High school	50	50	50	50

	Malaga	Cadiz
*University education*		
Social sciences	20	20
Humanities	20	20
Health sciences	20	20
Sciences	20	20
Engineering	20	20

Although the present study only analyses the data produced for the stimulus *movimientos feministas* ‘feminist movements’, the participants produced word lists for the following stimuli during the tests:

1. *inmigración* ‘immigration’
2. *movimientos feministas* ‘feminist movements’
3. *cambio climático* ‘climate change’
4. *animalistas* ‘animal activists’
5. *violencia de género* ‘gender-based violence’
6. *educación sexual* ‘sex education’
7. *la Constitución* ‘the Spanish Constitution’
8. *movimientos políticos populistas* ‘populist political movements’
9. *memoria histórica* ‘historical memory’
10. *independentismo* ‘separatism’

Participants had two minutes in each case to produce a word list. The lists were then combined for each stimulus and analysed as a whole to establish the community prototypes. In order to calculate the lexical compatibility of the terms provided and establish the different levels of compatibility, the *DispoCen* program was used. This tool was produced at the University of Malaga (Ávila-Muñoz et al., 2021) and consists of a library of functions in R that allow researchers to implement various models and applications, which include the option of instantly calculating the functionalities relating to availability and compatibility. The program can be accessed via the following link: https://github.com/jmss70/dispocen.

Participants also filled in a questionnaire that gathered information regarding their basic sociological characteristics as well as information regarding their immediate family’s social biography, the participant’s own leisure and cultural habits, their contact with the media and social media, their attitudes towards certain social and linguistic phenomena, their contact with technology, etc.

Since gender identity provided the most interesting prototypical conceptualisations in this study, Table 2 presents the distribution of the sample according only to this variable. One participant from Cadiz enrolled in high school did not provide information for gender identity, which is why Table 2 refers to a total of only 599 participants. Also, as can be observed, only four participants selected the option *Other*. Therefore, the present work focusses only on the differences between men and women regarding this variable.

**Table 2:** Distribution of the sample by gender identity

	Man	Woman	Other	Total
Entire sample	270	335	4	599
Malaga	135	165	0	300
Cadiz	135	160	4	299
Compulsory secondary education	100	97	3	200
High school	96	103	0	199
University	74	125	1	200

## Analysis and Results

As stated above, the results presented in this section were obtained using the tool *DispoCen*. Based on the individual lists of available vocabulary provided for the stimulus *movimientos feministas* ‘feminist movements’, *DispoCen* weighted the frequency of appearance of all the words provided and their positions on the lists. It then identified a certain set of data as prototypical and compared each additional element to this set to determine its degree of compatibility with the set. This process resulted in a model in which all words had been assigned an ILC between 0–1, with the value of 1 describing maximum compatibility. *DispoCen* then set the initial level of compatibility to consist of words presenting an ILC of 0.95299 or higher (Level 6, level of maximum compatibility, see Table 4) and determined cut-off marks for further levels of compatibility (Levels 5–1) that expanded progressively outwards and away from the nucleus; Level 1 encompassed the words that were least compatible with the community prototype.

### General Analysis

Table 3 presents the words that obtained the highest ILCs overall^1^: *right*, *equality*, *struggle*, *machismo*, *demonstration*, and *purple*. These constitute the general opinion shared by the sample with regard to feminist movements, since their high ILCs make them the most compatible within the lexical metastructure. They show that the students under study associate the phenomenon with the demands made by these movements as well as a spirit of struggle, giving the initial impression of an objective and positive shared perception.

**Table 3:** General analysis—words with the highest ILCs

Word	Level of compatibility	ILC	N
*derecho* ‘right’	6	0.98345	200
*igualdad* ‘equality’	6	0.98344	390
*lucha* ‘struggle’	6	0.98340	197
*machismo* ‘machismo’	6	0.98340	209
*manifestación* ‘demonstration’	6	0.98340	249
*morado* ‘purple’	6	0.98333	180

Table 4 presents all the words included in Level 6, the level of maximum compatibility. Certain words can now be observed that cause a need for reflection: *feminazi*, *hembrismo*, *radical*, *falseness*, *lie*, and *nonsense*. These words indicate a certain degree of dissatisfaction and, perhaps, a slightly distorted perception.

**Table 4:** Words included in the level of maximum compatibility

Level of compatibility	N	Words (ILC ≥ 0.95299)
6	65	*derecho* ‘right’, *igualdad* ‘equality’, *lucha* ‘struggle’, *machismo* ‘machismo’, *manifestación* ‘demonstration’, *morado* ‘purple’, *mujer* ‘woman’, *feminismo* ‘feminism’, ***feminazi* ‘feminazi’**, *huelga* ‘strike’, *desigualdad* ‘inequality’, *hombre* ‘man’, *8M* ‘8M’, *libertad* ‘freedom’, *protesta* ‘protest’, *violencia* ‘violence’, *violación* ‘rape’, *pancarta* ‘banner’, *ayuda* ‘help’, *injusticia* ‘injustice’, *justicia* ‘justice’, ***hembrismo* ‘hembrismo’**, *política* ‘politics’, *maltrato* ‘mistreatment’, *Podemos* ‘Podemos’, *fuerza* ‘force’, *poder* ‘power’, *8 de marzo* ‘8^th^ March’, *necesario* ‘necessary’, *muerte* ‘death’, *apoyo* ‘support’, *reivindicación* ‘recognition’, *feminista* ‘feminist’, *empoderamiento* ‘empowerment’, *género* ‘gender’, *respeto* ‘respect’, *patriarcado* ‘patriarchy’, *ley* ‘law’, *trabajo* ‘work’, *unión* ‘union’, *abuso* ‘abuse’, *cambio* ‘change’, *revolución* ‘revolution’, *discriminación* ‘discrimination’, *sociedad* ‘society’, *necesidad* ‘necessity’, *miedo* ‘fear’, *equidad* ‘fairness’, *superioridad* ‘superiority’, *movimiento* ‘movement’, ***radical* ‘radical’**, *acoso* ‘harassment’, *marcha* ‘march’, *violencia de género* ‘gender-based violence’,* (Irene) Montero* ‘(Irene) Montero’, *progreso* ‘progress’, *oportunidad* ‘opportunity’, *sororidad* ‘sorority’, ***falsedad* ‘falseness’**, ***mentira*** **‘lie’**, *cartel* ‘poster’, *calle* ‘street’, ***tontería*** **‘nonsense’**, *opresión* ‘oppression’, *salario* ‘salary’

### Analysis by Gender Identity

Table 5 presents the words that show the biggest differences when comparing the ILCs obtained for women and men.

**Table 5:** Comparison of ILCs for women and men

Word	ILC Women	ILC Men
*sororidad* ‘sorority’	0.96750332	0.05263158
*moda* ‘trend’	0.11111111	0.84444444
*fuerza* ‘force’	0.99083805	0.26050420
*niña* ‘girl’	0.89561993	0.18039216
*luchar* ‘fight’	0.88938272	0.18333333
*futuro* ‘future’	0.74768793	0.10000000
*familia* ‘family’	0.80931277	0.16666667
*seguridad* ‘security’	0.86032201	0.25333333
*asco* ‘disgust’	0.33333333	0.89062500
*feminicidio* ‘femicide’	0.74320988	0.20879121
*unión* ‘union’	0.99059714	0.48603441
*superioridad* ‘superiority’	0.46545455	0.94081439
*falsedad* ‘falseness’	0.50000000	0.93300781
*acoso* ‘harassment’	0.90616541	0.48729670
*(Irene) Montero* ‘(Irene) Montero’	0.53655435	0.94961271
*mentira* ‘lie’	0.53230769	0.87784864
*oportunidad* ‘opportunity’	0.90749642	0.60493827
*desinformación* ‘disinformation’	0.53962054	0.83428030
*salario* ‘salary’	0.54140305	0.82619114
*necesidad* ‘necessity’	0.96155695	0.67692308
*miedo* ‘fear’	0.95845208	0.68088643
*opresión* ‘oppression’	0.67329545	0.83858907

Note the words that are significantly more compatible in the case of women: *sorority*, *force*, *union*, *harassment*, *opportunity*, *necessity*, and *fear*. These all present ILCs of over 0.9 for women, while for men one of these does not even reach an ILC of 0.1 (*sorority*). Some of the words that present higher ILCs for men are also worth noting: *superiority*, *falseness*, *disgust*, *lie*, *trend*, and *disinformation*. The word *trend* in particular is much more compatible for men than women (0.84444444 versus 0.11111111), possibly suggesting that men consider feminism to be more of a passing trend than a historical endeavour

### Analysis by Educational Level

This section presents the results obtained according to the three educational levels considered in this work. Any differences observed here may be relevant, as they could indicate successes or failures regarding the subject of equality in current educational curricula.

#### Compulsory Secondary Education

For students enrolled in the fourth and final year of compulsory secondary education (*Educación Secundaria Obligatoria*), Table 6 presents the words obtaining the highest ILCs, while Table 7 presents the words included in all six levels of compatibility.

**Table 6:** Words with the highest ILCs—compulsory secondary education

Word	ILC	N
*igualdad* ‘equality’	1.000000	56
*mujer* ‘woman’	1.000000	59
*morado* ‘purple’	0.999999	45
*manifestación* ‘demonstration’	0.999993	49
*feminazi* ‘feminazi’	0.999973	27
*lucha* ‘struggle’	0.999970	39

**Table 7:** Words included in the different levels of compatibility—compulsory secondary education

Level of compatibility	N	Words
6	11	*igualdad* ‘equality’, *mujer* ‘woman’, *morado* ‘purple’, *manifestación* ‘demonstration’, *feminazi* ‘feminazi’, *lucha* ‘struggle’, *huelga* ‘strike’, *machismo* ‘machismo’, *derecho* ‘right’, *feminismo* ‘feminism’, *violencia* ‘violence’
5	2	*libertad* ‘freedom’, *8 M* ‘8 M’
4	5	*violación* ‘rape’, *Podemos* ‘Podemos’, *hombre* ‘man’, *desigualdad* ‘inequality’, *asco* ‘disgust’
3	8	*maltrato* ‘mistreatment’, *loca* ‘crazy’, *hembrismo* ‘hembrismo’, *muerte* ‘death’, *fuerza* ‘force’, *injusticia* ‘injustice’, *poder* ‘power’, *justicia* ‘justice’
2	15	*protesta* ‘protest’, *superioridad* ‘superiority’, *exageración* ‘exaggeration’, *8 de marzo* ‘8th March’, *abuso* ‘abuse’, *revolución* ‘revolution’, *radical* ‘radical’, *pancarta* ‘banner’, *trabajo* ‘work’, *antifeminismo* ‘antifeminism’, *empatía* ‘empathy’, *ayuda* ‘help’, *política* ‘politics’, *unión* ‘union’, *debate* ‘debate’
1	31	*falsedad* ‘falseness’, *pelo en sobaco* ‘armpit hair’, *respeto* ‘respect’, *cartel* ‘poster’, *discriminación* ‘discrimination’, *empoderamiento* ‘empowerment’, *barco* ‘boat’, *derechos de la mujer* ‘women’s rights’, *desvío* ‘deviation’, *en contra del maltrato* ‘against mistreatment’, *ilegal* ‘illegal’, *inutilidad* ‘uselessness’, *luchar por los derechos de la mujer* ‘to fight for women’s rights’, *no a la violencia* ‘say no to violence’, *positivo* ‘positive’, *propaganda* ‘propaganda’, *quema* ‘burning’, *feminista* ‘feminist’, *insulto* ‘insult’, *tinte de pelo* ‘hair dye’, *grito* ‘shout’, *locura* ‘madness’, *reivindicación* ‘recognition’, *asesinato* ‘murder’, *acoso* ‘harassment’, *lila* ‘lilac’, *género* ‘gender’,* (Irene) Montero* ‘(Irene) Montero’, *violencia de género* ‘gender-based violence’, *agresión* ‘aggression’, *voto* ‘vote’

Tables 6 and 7 corroborate the initial impressions gained in the general analysis: although the shared perception is positive (*equality*, *woman*, *purple*, *struggle*), other words appear that could suggest a slight distortion (*feminazi* in Level 6 with an ILC of 0.999999, as well as *disgust*, *crazy*, and *hembrismo* in Levels 4 and 3, which are still very compatible).

**Fig. 1:**
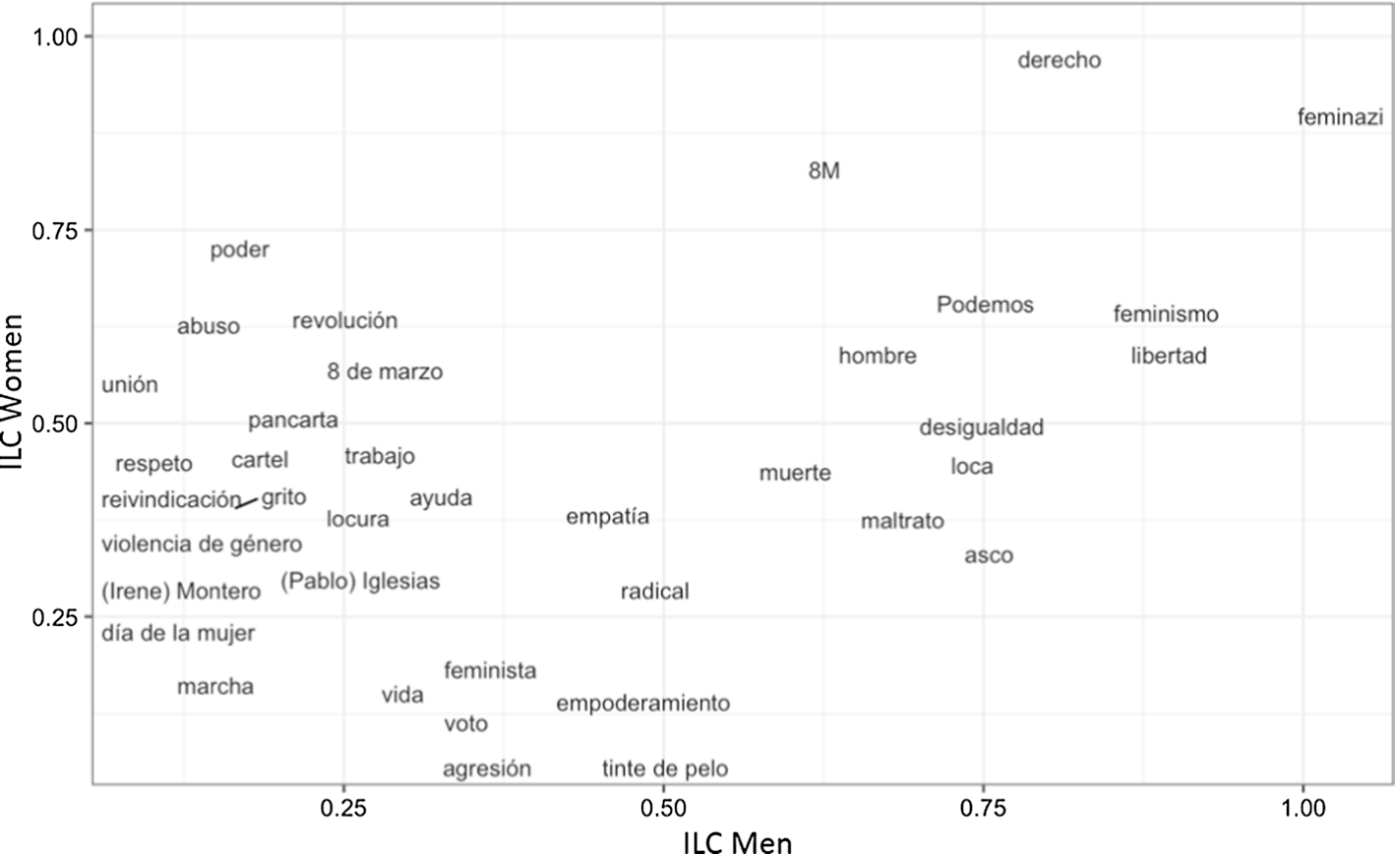
ILC. Comparison between men and women enrolled in compulsory secondary education

Figure 1 compares the results obtained for men and women at this educational level. Recall that the words with ILCs closest to 1 are the most compatible in the case of each group. Table 8 contains the translations of the words appearing in Fig. 1.

**Table 8:** Translations of the words appearing in Fig. 1

Words	ILC Women	ILC Men
*poder* ‘power’	0.69696970	0.14141414
*unión* ‘union’	0.55555556	0.07142857
*abuso* ‘abuse’	0.60000000	0.14285714
*asco* ‘disgust’	0.33333333	0.78125000
*revolución* ‘revolution’	0.58333333	0.22857143
*empoderamiento* ‘empowerment’	0.12500000	0.46666667
*libertad* ‘freedom’	0.54518950	0.86666667
*agresión* ‘aggression’	0.06666667	0.37500000
*respeto* ‘respect’	0.44444444	0.14285714
*loca* ‘crazy’	0.40000000	0.70000000
*tinte de pelo* ‘hair dye’	0.11111111	0.40740741
*8 de marzo* ‘8^th^ March’	0.56709957	0.27272727
*feminismo* ‘feminism’	0.65909091	0.93925000
*muerte* ‘death’	0.38296399	0.65820312
*cartel* ‘poster’	0.44973545	0.17647059
*pancarta* ‘banner’	0.51785714	0.25000000
*maltrato* ‘mistreatment’	0.40923077	0.67619048
*feminista* ‘feminist’	0.14285714	0.40740741
*8M* ‘8M’	0.83163265	0.56709957
*reivindicación* ‘recognition’	0.38750000	0.12500000
*desigualdad* ‘inequality’	0.53714286	0.76136364
*violencia de género* ‘gender-based violence’	0.34375000	0.12500000
*voto* ‘vote’	0.12500000	0.33333333
*grito* ‘shout’	0.37000000	0.16304348
*trabajo* ‘work’	0.45142857	0.25925926
*ayuda* ‘help’	0.45299145	0.26530612
*radical* ‘radical’	0.33333333	0.50000000
*(Irene) Montero* ‘(Irene) Montero’	0.32027972	0.16666667
*vida* ‘life’	0.14285714	0.28571429
*hombre* ‘man’	0.57013567	0.71279121
*día de la mujer* ‘Women’s Day’	0.25000000	0.11111111
*locura* ‘madness’	0.33333333	0.20000000
*Podemos* ‘Podemos’	0.61038961	0.73333333
*derecho* ‘right’	0.97420268	0.86000000
*(Pablo) Iglesias* ‘(Pablo) Iglesias’	0.27572016	0.16666667
*marcha* ‘march’	0.20879121	0.10000000
*empatía* ‘empathy’	0.33333333	0.43722944

Overall, at this educational level, the prominent position of the word *disgust* among men is significant, as is the apparent association among women of the feminist movements with a spirit of protest (*union*, *revolution*, *8th March*, *banner*).

#### High School

For students enrolled in the second and final year of high school (*Bachillerato*), Table 9 presents the words obtaining the highest ILCs. In contrast to the previous educational level, this group’s shared perception now no longer includes pejoratives, with *feminazi* having been replaced by *right*.

**Table 9:** Words with the highest ILCs—high school

Word	ILC	N
*igualdad* ‘equality’	0.95019	141
*mujer* ‘woman’	0.95019	129
*machismo* ‘machismo’	0.95019	79
*derecho* ‘right’	0.95019	74
*morado* ‘purple’	0.95019	72
*manifestación* ‘demonstration’	0.95019	86

**Fig. 2:**
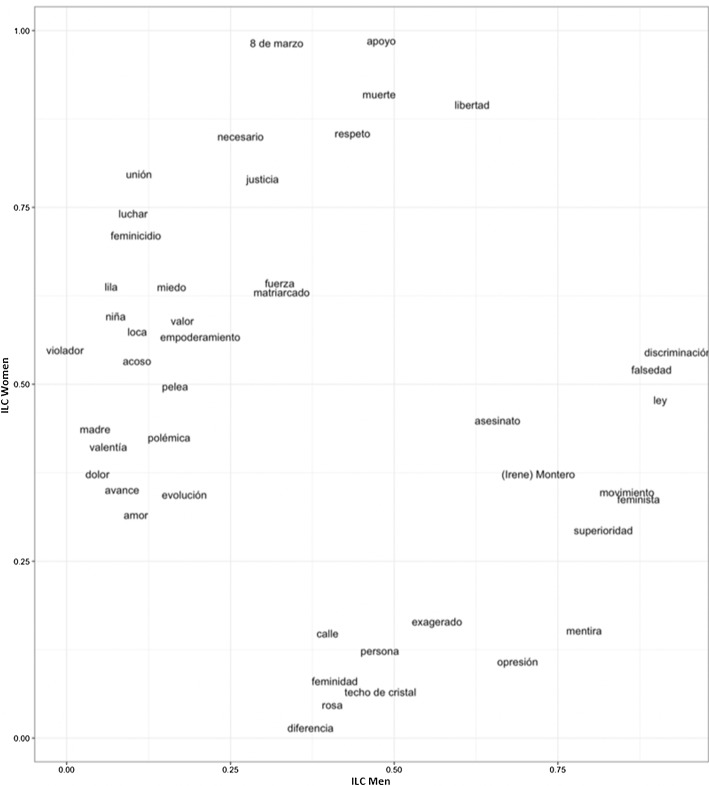
ILC Men and women enrolled in high school

Figure 2 shows that the word *feminazi* has now disappeared entirely, even specifically for men, although high ILCs for men are instead obtained by the words *lie* and *falseness*. For women, these two words present lower ILCs, and words associated with the act of protesting again predominate (*to fight*, *force*, *union*, *empowerment*, *fight*). As above, Table 10 contains the translations of the words appearing in Fig. 2.

**Table 10:** Translations of the words appearing in Fig. 2

Words	ILC Women	ILC Men
*mentira* ‘lie’	0.20000000	0.85905612
*feminista* ‘feminist’	0.31818182	0.93333333
*luchar* ‘to fight’	0.67592593	0.06666667
*niña* ‘girl’	0.65909368	0.05882353
*lila* ‘lilac’	0.67790850	0.08333333
*8 de marzo* ‘8th March’	0.98526936	0.39250000
*unión* ‘union’	0.73529412	0.14438503
*movimiento* ‘movement’	0.32692308	0.87529617
*miedo* ‘fear’	0.67312721	0.14285714
*feminicidio* ‘femicide’	0.66666667	0.14285714
*muerte* ‘death’	0.96433421	0.45032051
*necesario* ‘necessary’	0.81971154	0.32500000
*respeto* ‘respect’	0.87935553	0.40000000
*opresión* ‘oppression’	0.17857143	0.65777778
*violador* ‘rapist’	0.50769231	0.04761905
*apoyo* ‘support’	0.93146261	0.49375000
*madre* ‘mother’	0.48235294	0.04545455
*valor* ‘courage’	0.52361111	0.10000000
*exagerado* ‘exaggerated’	0.12500000	0.53333333
*discriminación* ‘discrimination’	0.48788294	0.89096154
*justicia* ‘justice’	0.74389756	0.34375000
*superioridad* ‘superiority’	0.37000000	0.76190476
*matriarcado* ‘matriarchy’	0.63839286	0.25000000
*(Irene) Montero* ‘(Irene) Montero’	0.31818182	0.70055556
*fuerza* ‘force’	0.64102564	0.26050420
*ley* ‘law’	0.54662915	0.92156863
*loca* ‘crazy’	0.50000000	0.14285714
*empoderamiento* ‘empowerment’	0.60204082	0.25000000
*acoso* ‘harassment’	0.49473684	0.14285714
*falsedad* ‘falseness’	0.50000000	0.83593750
*polémica* ‘controversy’	0.50000000	0.16666667
*techo de cristal* ‘glass ceiling’	0.10256410	0.42591250
*feminidad* ‘femininity’	0.04000000	0.35714286
*persona* ‘person’	0.08333333	0.39843750
*diferencia* ‘difference’	0.07692308	0.38888889
*avance* ‘advance’	0.36805556	0.05882353
*pelea* ‘fight’	0.54962963	0.24210526
*calle* ‘street’	0.16923077	0.47500000
*dolor* ‘pain’	0.39738292	0.10526316
*valentía* ‘bravery’	0.41538462	0.12550607
*amor* ‘love’	0.37777778	0.09569378
*asesinato* ‘murder’	0.39235165	0.66911765
*libertad* ‘freedom’	0.96226797	0.68750000
*rosa* ‘pink’	0.06250000	0.33629630
*evolución* ‘evolution’	0.39047619	0.12500000

#### University

The results obtained for students enrolled in the final year of a bachelor’s degree (*Grado Universitario*) confirm the tendencies identified above. As can be observed in Table 11, the words presenting the highest ILCs for this group relate only to equal rights, the main demand of feminist movements.

**Table 11:** Words with the highest ILCs—university

Word	ILC	N
*igualdad* ‘equality’	1.0000000	139
*mujer* ‘woman’	1.0000000	110
*derecho* ‘right’	1.0000000	68
*lucha* ‘struggle’	1.0000000	76
*machismo* ‘machismo’	0.9999999	77
*manifestación* ‘demonstration’	0.9999940	80

Even if the entire level of maximum compatibility for students at this educational level is observed instead (Table 12), then apart from *feminazi*, all words still refer either to legitimate causes supported by these movements (*equality*, *right*, *freedom*) or to inequities that warrant their existence (*machismo*, *inequality*, *patriarchy*, *violence*).

**Table 12:** Words included in the level of maximum compatibility—university

Level of compatibility	N	Words
6	24	*igualdad* ‘equality’, *mujer* ‘woman’, *derecho* ‘right’, *lucha* ‘struggle’, *machismo* ‘machismo’, *manifestación* ‘demonstration’, *feminismo* ‘feminism’, *morado* ‘purple’, *libertad* ‘freedom’, *hombre* ‘man’, *desigualdad* ‘inequality’, *necesario* ‘necessary’, *empoderamiento* ‘empowerment’, *feminazi* ‘*feminazi’*, *política* ‘politics’, *patriarcado* ‘patriarchy’, *justicia* ‘justice’, *violencia* ‘violence’, *8 M* ‘8 M’, *necesidad* ‘necessity’, *ayuda* ‘help’, *oportunidad* ‘opportunity’, *poder* ‘power’, *unión* ‘union

#### *Feminazi*—A Cause for Concern?

The appearance of words that could indicate distorted perceptions of the feminist movements is a cause for concern. Words such as *feminazi*, *hembrismo*, *crazy*, and *lie* suggest a need for further analysis. As the word *feminazi* appeared in the level of maximum compatibility in the general analysis and also for several of the groups analysed thereafter, the more detailed analysis conducted in response to the results obtained above and presented in this section focusses on this word as a paradigm for the others. An additional dataset was produced that describes the positions obtained by the word *feminazi* on the individual lists, and two figures were created that combine this data with sociological information on the participants that had the potential to be of relevance.

**Fig. 3:**
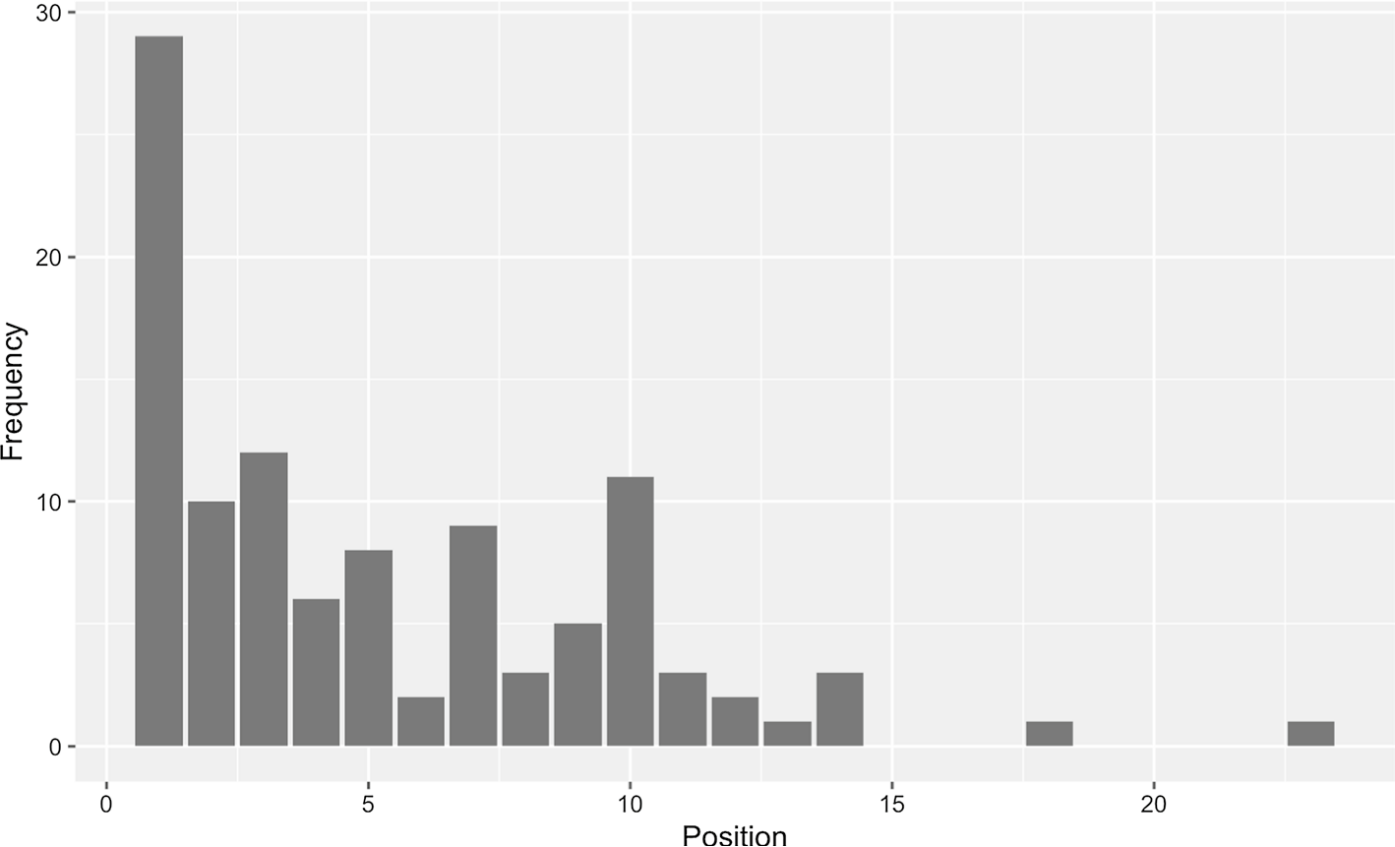
Histogram of position and frequency of appearance of the word feminazi

The word *feminazi* was provided by 106 participants out of the total sample (N = 600). Figure 3 presents the positions in which the word appeared on the lists of these individuals.

**Fig. 4:**
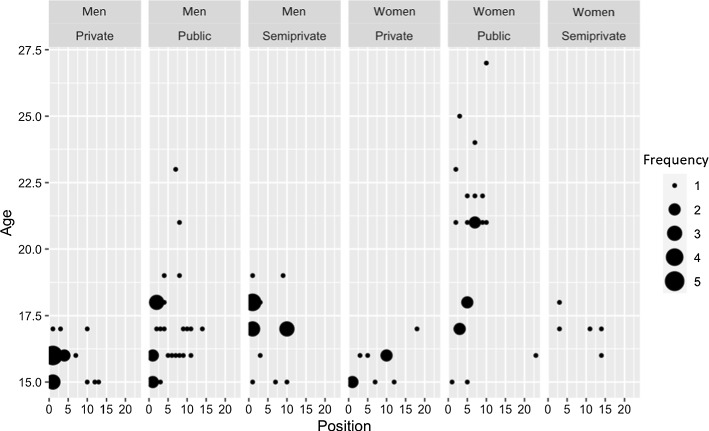
Appearance of the word feminazi according to age, gender identity, and type of centre (I)

**Fig. 5:**
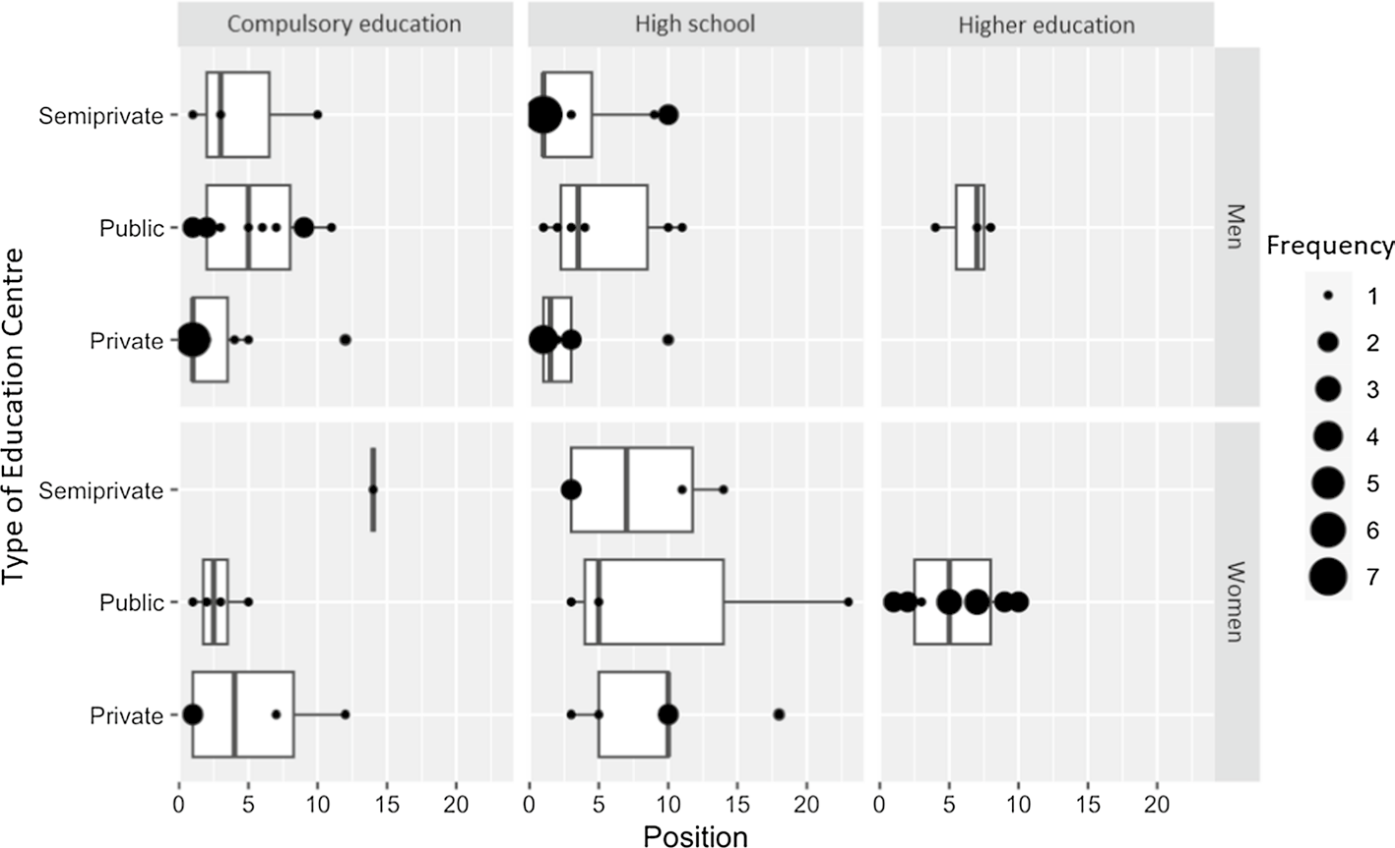
Appearance of the word feminazi according to age, gender identity, and type of centre (II)

Note that even though only just over a sixth of all participants provided the word, most of these individuals provided it in first position. This explains the high ILC identified in the general analysis. Figures 4 and 5 present the associations observed between the appearance of *feminazi* and the sociological characteristics of the individuals who provided the word.

Overall, the word *feminazi* appears in higher positions on the lists provided by men, and it appears more frequently among students enrolled at private and semi-private (state-subsidised) centres. Although linear regression analysis did not show a statistically significant correlation, the tendencies identified in this section nevertheless warrant contemplation and should be noted.

## Discussion

The general aim of this work was to study the collective perception held by a sample of Spanish students regarding feminist movements. Additional, more detailed aims included observing how sociocultural factors affect this perception, conducting lexical analysis, and analysing the cognitive prototypes present in the collective mental representation produced by the study sample for the phenomenon. The ultimate aim of this work was to identify areas of perception that may have been recently acquired by society or may even be discriminatory towards present-day social movements that seek to achieve gender equality. In order to achieve these aims, a sample of 600 students from the provinces of Malaga and Cadiz, Spain, took part in tests of lexical availability. The procedures used for these association tests have been widely used in the fields of sociolinguistic and psycholinguistic research. Despite their simplicity, they have proven to be an incredibly useful method for obtaining large databases, hence it is this form of data that was used for the analysis. The data was then analysed using a novel approach that incorporates fuzzy set theory into the traditional framework of lexical availability, allowing for the objective construction of collective prototypes. The possible distortions identified in the shared perceptions seem to reflect the results of other studies, discussed below.

The situation in Spain regarding gender equality reflects that in today’s democratic societies described in the Introduction. At the institutional level, various laws aim to counteract, eliminate, and prevent any form of discrimination based on gender identity (e.g., Spanish Organic Law 3/2007 of 22 March for Effective Equality Between Women and Men, and Spanish Organic Law 4/2007 of 12 April on Universities). However, the population does not seem entirely aligned with this goal, as shown below. If one looks beyond the financial costs of implementing policies that ultimately appear to be unsuccessful and considers the words of Amelia Valcárcel (2008, p. 75), “una democracia, cuando funciona, es feminista” (‘a democracy, when it works, is feminist’), the situation both in Spain and in other democratic societies could in fact indicate that these democracies are not functioning as they should.

According to data published by the Spanish Centre of Sociological Research (CIS, 2014), less than 6% of young people in Spain label themselves as feminist. Regarding university students specifically, Velasco Martínez (2016) demonstrated an alarming lack of information on feminism among this section of society and observed that individuals cannot be expected to act in a manner suited to a situation they do not fully understand. The author concluded that young people must be made aware of gender-based discrimination through education, stating that if students can recognise gender-based discrimination, this may facilitate feminist identification. Weis et al. (2018) came to a similar conclusion in their study of 428 U.S. women, in which they suggested that informing women of the pervasiveness of sexism could promote feminist identification.

Velasco Martínez (2016) also noted the system errors and highlighted that patriarchal patterns as well as the traditionalism imposed by religion and politics were preventing feminist movements from being accepted by the Spanish university population, the subject of the author’s research. Moreover, even among those students that did identify with feminist values, feminist labelling was hampered by student’s perceiving a close link between feminism and political and radical social activism (even though it may be the case that this link is in fact promoted precisely by the neoliberal and patriarchal ideology).

In terms of the research presented in this work, the concerning distortions identified in the analysis could be mirroring a similar degree of disinformation with regard to feminist movements among the student population under study. The systematic and almost universal appearance of the word *feminazi* attracted attention, as it could indicate the presence within this population of a concerning perception of feminism that can also be widely observed at the global level within some sections of society, including among certain women (Swirsky & Angelone, 2014). One reason given by Swirsky and Angelone (2014) for resentment among women was “a negative connotation associated with the term ‘feminist’”. In their study of just under 500 women residing in the United States, practically half who rejected a feminist identity did so due to the frequent association of feminism with radical factions and the overall stigma surrounding feminism. These authors noted that, as is the case for all social movements, extreme factions of feminism do exist. They stated that certain media outlets may focus on these more radical groups, presenting them in a way intended to make such attitudes seem widespread in certain sections of society and possibly causing a general distaste for the entire movement. In future research, it would be interesting to determine what impression of the feminist movements is being endorsed at public and private/semi-private education centres, so as to identify whether the possible distortions observed in the present work may have been acquired by students within the classroom.

## Conclusions

This work analysed lists of lexical association produced for the stimulus *movimientos feministas* ‘feminist movements’ by a sample of 600 Spanish students from Malaga and Cadiz, enrolled in the final year of compulsory secondary education (*Educación Secundaria Obligatoria*), the final year of high school (*Bachillerato*), and the final years of various bachelor’s degrees (*Grados Universitarios*) across all fields of knowledge. For the non-university levels, data was gathered from both public and private/semi-private (state-subsidised) centres. The analysis was based on an approach and calculation that produces each word’s index of lexical compatibility, a value that compares each word with those that are initially identified as being most compatible within the centre of interest. The two cornerstones of the approach were categorisation theory and fuzzy set theory, and although these have a strong presence respectively in the areas of psycholinguistics and mathematics, the model described in this work involves a methodology that combines the two theories for the first time. This original methodology made it possible for the present research to access the perceptions and associated opinions held by the study population in connection with the phenomenon of feminism.

Most of the previous studies based on lexical association tests—especially those from the field of lexical availability—do not usually include initial stimuli of social relevance or impact. With our work, we show that including such stimuli may be of interest for psycho-sociolinguistic research. It has become clear that the concrete results of working with the methodology proposed here have an obvious application in certain socially sensitive areas. Thanks to this methodological innovation, we can better understand and propose improvement plans for problems affecting socially vulnerable groups.

This study contributes to advancing knowledge of Spanish students’ perceptions of the phenomenon of feminism and adds originality by offering a different approach to previous studies based on reproducibility, replicability, and reliability. By providing a solid and reliable basis for future research, it positions itself as a key reference in the scientific field and provides a solid starting point to continue exploring this important social issue.

**Fig. 6:**
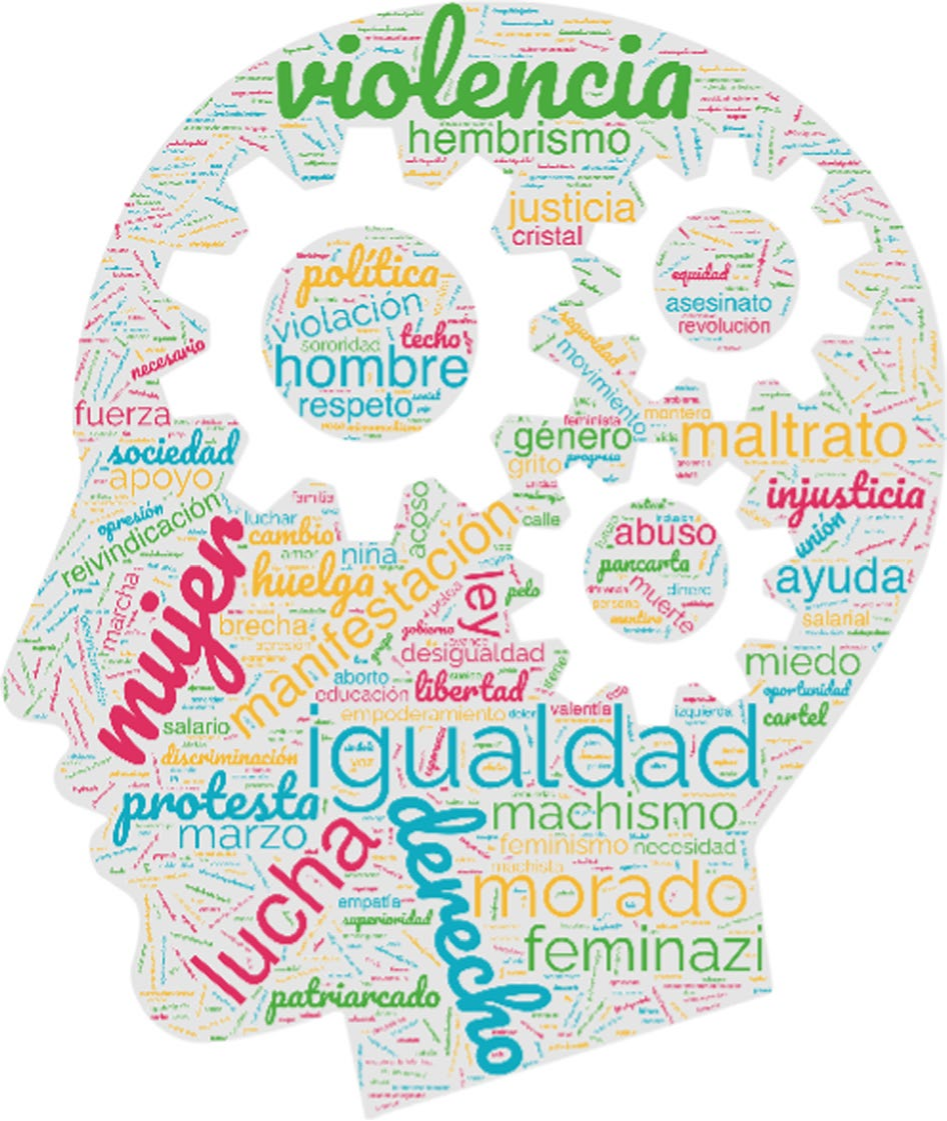
Visual representation of the shared categorisation for feminist movements

Overall, the results obtained are promising. As can be observed in Fig. 6, the sample under study produced a mostly positive shared categorisation that reflects the values of equality so characteristic of the feminist movements throughout history.

The words repeated most often and that best reflect the shared perception of feminist movements are: *derecho* ‘right’, *igualdad* ‘equality’, *lucha* ‘struggle’, *machismo* ‘machismo’*, manifestación* ‘demonstration’, *morado* ‘purple’, and *mujer* ‘woman’. Other very compatible and representative words refer to the spirit of protest for which these movements are known today: *huelga* ‘strike’, *desigualdad* ‘inequality’, *8M* ‘8M’, *libertad* ‘freedom’, *protesta* ‘protest’, *pancarta* ‘banner’, *reivindicación* ‘demand’, *unión* ‘union’, *revolución* ‘revolution’, *marcha* ‘march’, *sororidad* ‘sorority’, *cartel* ‘poster’, and *calle* ‘street’. Nevertheless, among the words that were most compatible with the shared categorisation, certain words attracted attention: *feminazi* ‘feminazi’, *hembrismo* ‘hembrismo’, *radical* ‘radical’, *falsedad* ‘falseness’, *mentira* ‘lie’, and *tontería* ‘nonsense’. Due to the high ILCs of some of these words and their repeated appearance on the lists under study, additional analysis was conducted focussed on *feminazi*, the word that may best represent the values that could be implied by them all. The results of this more detailed analysis showed a noteworthy tendency of this word to be provided more often by men than women, and more often by individuals enrolled at private/semi-private centres than individuals enrolled at public centres.

Although the analysis did record an association with politics in higher levels of compatibility, with individuals having provided words such as *politics*, *Podemos*, and *(Irene) Montero*, the most frequent direct associations with the feminist movements were the words *necessary*, *support*, *empowerment*, *union*, *change*, *society*, *necessity*, *fairness*, *progress*, *opportunity*, and *girl*. These suggest a promising future for society in which men and women could at last have equal rights.

As can be observed, however, the cognitive gaps as well as the more recently acquired perceptions identified in the students under study here could possibly be in line with the results of previous studies regarding the social spread of feminism and the creation of more equitable societies. For this reason, I—the author of this work—similarly suggest that society could perhaps achieve an increase in students’ awareness of the societal risks posed by discriminating against women if classrooms at the various levels of education both in public and private/semi-private centres shared a larger amount of objective and substantiated information on the feminist movements and the causes they represent. The following list provides specific suggestions that could be implemented at education centres and that could potentially help prevent discrimination against women:

1. *Implementation of awareness programmes at early stages of education* Targeted, awareness-raising programmes directed at children could be designed for early levels of education. These would need to address misconceptions and facilitate a more complete and realistic understanding of feminist movements.
2. *Inclusion of feminist movements in the school curriculum* Including education on the feminist movements in the school curriculum would allow issues relating to gender equality and the movements themselves to be incorporated into the classroom at early ages. This could help to counteract stereotyping and prejudice regarding feminist movements.
3. *Use of inclusive activities* Inclusive classroom activities could be organised to encourage the active participation of all students, regardless of gender. This could involve talks, projects, and discussions on gender equality.

I also recommend that education may wish to focus on students who identify as women and on demonstrating their personal vulnerability to such discrimination. This focus could encourage this group of individuals to label themselves actively and unequivocally as feminists, so that they may then help their fellow students of other genders to accept feminist movements as a necessary component for creating fairer societies and to adopt a feminist awareness distanced from extreme ideologies. This approach might once and for all make it possible for society to overcome the historic social issue of gender inequality.

Although this study provides valuable information regarding Spanish student’s perceptions of the feminist movements, it does exhibit certain limitations. First, the sample size of 600 students may not reflect all the opinions held by the entire student populations in the cities under study. A larger sample size would be required to provide a better reflection. Second, using association tests to construct the shared metastructure on which analysis is then based may simplify the complexity of the individuals’ beliefs. Complementary tests in the form of focus groups on the subject of feminism are currently being prepared to obtain more detail. Third, the recommendation that education centres may wish to share a larger amount of objective information with students assumes a shared understanding of what constitutes “objective” information on the feminist movements. Collaboration with specialists would be required to ensure this shared understanding.

Regarding directions for future research, since society consists of many more environments than those observed here and gender inequality is present across the board, further analyses along the same lines as described herein but focussing on other sections within society is required. In addition, the focus groups currently being prepared to corroborate the results obtained above could also be used to allow students to freely express their views regarding other issues apart from gender equality, such as gender-based violence, sex education, and other issues that would help to determine the directions to take in future work.

## Data Availability

The dataset generated for this study includes data originating from underage individuals and is not publicly available as it contains proprietary information that the author acquired through a licence. Due to privacy concerns and the need to protect the identities of minors, access to the dataset and instructions for reproducing the analysis can be obtained by contacting the author directly.

## Footnotes

1 Items within the text will be presented in translation only. Items within tables and figures will be presented in their original form together with their translation.
